# The monocyte to high-density lipoprotein cholesterol ratio is a risk factor for frequent premature ventricular complexes: a retrospective cohort study

**DOI:** 10.1186/s12944-022-01742-7

**Published:** 2022-12-03

**Authors:** Yunfei Wang, Deming Li, Xuetao Zhu, Jing Li, Cui Yue, Ling Wu, Qingqing Zhuan, Xiaomeng Dou, Wei Duan

**Affiliations:** 1 Department of Cardiology, HHCH, Hefei, Anhui China; 2 Department of Cardiology, FAHAMU, Hefei, Anhui China; 3 Department of Medical Records, HHCH, Hefei, Anhui China

**Keywords:** MHR, PVCs, ABC-VT risk score

## Abstract

**Background:**

Little is known about the link between the monocyte to high-density lipoprotein cholesterol ratio (MHR) and frequent premature ventricular complexes (PVCs). This investigation aimed to evaluate the link between the MHR and frequent PVCs in patients, as well as their outcomes, using the axis, burden, coupling interval–ventricular tachycardia (ABC-VT) risk score (ARS).

**Methods:**

Two hundred patients with frequent PVCs and 70 controls were retrospectively enrolled, and their general data were gathered. The MHR and ARS were calculated. Then, patients developing frequent PVCs were classified into a medium−/high-risk subgroup and a low-risk subgroup according to ARS. The results were evaluated employing comparative statistical analyses, Spearman’s correlation, logistic regression analyses, and receiver operating characteristic (ROC) curves.

**Results:**

The MHR in the controls was obviously lower than that in the frequent PVC group. In addition, the MHR was the lowest in the control group and highest in the medium−/high-risk subgroup, with that of the low-risk subgroup falling in the middle. Spearman’s correlation analyses showed that the MHR was positively correlated with the ARS (*ρ* = 0.307, *P* < 0.001). Ultimately, the MHR was found to be a risk factor for frequent PVCs in the multivariate analysis. In addition, an MHR cutoff point of 254.6 featured 67.50% sensitivity and 67.14% specificity for predicting frequent PVCs, and the area under the curve (AUC) reached 0.694 (95% confidence interval: 0.623–0.766) (*P* < 0.001).

**Conclusions:**

The MHR is positively and independently correlated with frequent PVCs and can be used as a practical, cost-saving and simple biomarker of inflammation owing to its value in predicting frequent PVCs. In addition, the MHR is crucial to risk stratification and prognosis, which may give it clinical value in the prevention and management of frequent PVCs.

## Background

Premature ventricular complexes (PVCs) denote any cardiac rhythm, including single complex or multiple consecutive rhythms, originating from a site below the atrioventricular node. Studies have revealed that the aggravation of heart failure and damage to left ventricular function are associated with highly frequent PVCs [1, 2]. The precise etiologies of PVCs are still unclear, but inflammatory states following cardiac surgery, pericarditis and myocarditis are frequently related to PVCs. Furthermore, evidence has evaluated that inflammation remains a high priority in the pathological process of arrhythmia diseases such as atrial fibrillation (AF) [3–9] and PVCs [10, 11]. Therefore, the specific relationship between inflammation and PVCs deserves more research.

In bone marrow, monocytes develop from their precursors and are then released into the circulation, where they enter tissues at sites of inflammation and discharge proinflammatory cytokines; thus, monocytes impact inflammation severity and are regarded as a biomarker of inflammation [12]. High-density lipoprotein cholesterol (HDL-c), which relies on the reversal of low-density lipoprotein cholesterol (LDL-c) oxidation, transports cholesterol from the surrounding tissue to the liver for recycling and suppresses endothelial adhesion molecule expression as well as monocyte recruitment in the arterial wall, thus playing an antioxidant role and modulating thrombosis, vascular motor function and vascular inflammation [13–15]. The above studies found that by directly acting on monocytes, HDL-c can inhibit inflammatory responses. Given that the monocyte to HDL-c ratio (MHR) is related to the proinflammatory effects from monocytes and the anti-inflammatory effects from HDL-c, it is regarded as a neotypic marker of inflammation [16].

An increased MHR has been shown to be related to multiple diseases, such as metabolic syndrome [17], polycystic ovary syndrome [18], coronary artery disease [16, 19], and AF [20]. However, few studies have been carried out to evaluate the link between the MHR and frequent PVCs or patient outcomes.

The axis, burden, coupling interval–ventricular tachycardia (ABC-VT) risk score (ARS) was derived and verified by Voskoboinik et al. for distinguishing persons with frequent PVCs who are at higher risk of negative events; using the British Columbia PVC Registry population, external validation of the ARS was completed by Thibert et al. [21, 22]. Di Biase et al. analyzed Voskoboinik’s paper in detail and hypothesized that the score could guide clinicians in the management of persons with frequent PVCs [23]. Therefore, the ARS could be crucial to the prognosis of persons with frequent PVCs and could provide a reference for further clinical treatment options.

This investigation intends to explore the link between the MHR and frequent PVCs in patients, as well as their outcomes, using the ARS.

## Methods

### Study population

Between January 2018 and July 2022 in Hefei, China, 200 hospitalized persons who developed frequent PVCs [burden > 5%, left ventricular ejection fraction (LVEF) > 45%], aged 7–88 years, were recruited from the Hefei High-tech Cardiovascular Hospital (HHCH) and the First Affiliated Hospital of Anhui Medical University (FAHAMU). These persons had no structural heart diseases. Seventy hospitalized patients without frequent PVCs were selected as the controls. The exclusion criteria were as follows: recent blood transfusion, hematological disorder, autoimmune diseases, active infection, active or chronic inflammation, acute or chronic renal/hepatic diseases, and malignancy. The ethics committees of HHCH and FAHAMU approved the study protocol.

The ARSs were independently determined by two cardiologists, who were blinded to the data of each case. When the scores determined by the two cardiologists were inconsistent, the data were handed over to a third cardiologist, who made the decision. As Voskoboinik et al. described, the calculation standard of ARS is as follows: 4 points, a PVC coupling interval > 500 ms; 4 points, a nonsustained ventricular tachycardia; 3 points, a PVC burden > 20%; 2 points, a PVC burden 10 to 20%; 1 point, a superior PVC axis. On the basis of the ARS, the participants were divided into a low-risk subgroup (0–4 points) and a medium−/high-risk subgroup (5–12 points).

### Definitions

The general data of each person, including medication use, left ventricular end-diastolic (LVD) diameter (LVDD), LVEF, history of smoking and alcohol consumption, diabetes, hypertension, body mass index (BMI), sex and age, were gathered after admission. Person blood pressure, height and weight were determined thrice using standard techniques, and the three measurements were averaged as the final values. BMI was computed via dividing body weight (kg) by height (m) square. Hypertension referred to diastolic blood pressure (BP) ≥90 mmHg and/or systolic BP ≥140 mmHg without the use of antihypertensive drugs. Diabetes referred to a 2-hour blood glucose (BG) ≥200 mg/dl in an oral glucose tolerance test or a fasting BG ≥126 mg/dl without the use of oral hypoglycemic drugs or insulin. LVDD was recorded as the mean of values measured in five consecutive cardiac cycles, and LVEF was computed from the formula [LVEF = (left ventricular stroke volume/LVD volume)*100%], determined by one experienced doctor specializing in echocardiography.

### Blood analysis methods

Peripheral venous blood specimens were collected from participants upon admission to an inpatient ward. In all participants, blood tests were performed immediately after admission. White blood cell (WBC), monocyte, neutrophil, and lymphocyte counts and triglyceride, HDL-c, total cholesterol (TC), and LDL-c levels were measured. Subsequently, the MHR was calculated. A Sysmex XN-9000 automatic biochemical analyzer (Sysmex, Kobe, Japan) and a Zybio ZS400 automatic biochemical analyzer (ZYBIO Biotechnology Co., LTD, Chongqing, China) were applied to measure triglycerides, HDL-c, TC, and LDL-c. WBC, neutrophil, lymphocyte and monocyte counts were assessed by a DYMIND DT-CRP analyzer (DYMIND Biotechnology Co., LTD, Shenzhen, China) and a Beckman Coulter AU5800 analyzer (Beckman Coulter, California, USA).

### Statistical analyses

Variates are depicted as means ± standard deviations, and categorical variates are depicted as numbers (%). Disparities in features between the control and the frequent PVC group were tested by the t test, and the differences in the monocyte count, MHR, and HDL-c level between the ARS risk-stratified subgroups and control group were computed using one-way ANOVA followed by Tukey detection for multiple comparisons. To evaluate the associations of monocyte count, MHR, and HDL-c level with ARS, Spearman’s correlation analyses was applied. To evaluate the best cutoff values of the ARS and the specificity and sensitivity for all variables to predict frequent PVCs, receiver operating characteristic (ROC) analyses were applied. Last, binary logistic regression analyses (univariate and further multivariate) were applied to assess the risk factors (RFs) of frequent PVCs.

Each statistical analysis was conducted using GraphPad Prism 8.0 and SPSS 22.0. *P* < 0.05 was considered significant.

## Results

### Comparisons of baseline features between the control and frequent PVC groups

Table 1 showed the comparisons of baseline features between the control and frequent PVC groups. Persons in the frequent PVC group were younger and less likely to have a history of hypertension than persons in the control group. This study also found that LVDD, MHR, monocyte count, and lymphocyte count were higher in the frequent PVC group than the control group, but HDL-c and LVEF showed the opposite trend. More persons in the control group used statin medications, and more persons in the frequent PVC group used aspirin. The two groups exhibited obvious similarities in BMI, diabetes, smoking, alcohol consumption, WBC count, neutrophil count, triglyceride, TC, LDL-c, and angiotensin receptor blocker (ARB) or angiotensin-converting enzyme inhibitor (ACEI) medication use.

**Table 1:** Comparisons of baseline features between the frequent PVC and control groups

Features	Control group (***n*** = 70)	Frequent PVC group (***n*** = 200)	t/X^**2**^	***P***
**Demographic and clinical features**				
Male sex, n (%)	25.00(35.70)	84.00(42.00)	0.851	0.356
Age (y)	56.57 ± 15.59	51.34 ± 16.60	2.307	0.022
BMI (kg/m^2^)	24.14 ± 4.06	24.21 ± 3.80	−0.123	0.902
Hypertension, n (%)	31.00(44.30)	58.00(29.00)	5.483	0.019
Diabetes, n (%)	5.00(7.10)	12.00(6.00)	0.115	0.735
Smoking, n (%)	8.00(11.40)	24.00(12.00)	0.032	0.857
Alcohol consumption, n (%)	3.00(4.30)	16.00(8.00)	1.093	0.296
**Clinical and biochemical indexes**				
WBC count (× 10^9^/L)	5.52 ± 1.53	5.88 ± 1.54	−1.650	0.100
Neutrophil count (×10^9^/L)	3.36 ± 1.28	3.42 ± 1.20	−0.333	0.739
Lymphocyte count (×10^9^/L)	1.75 ± 0.56	1.93 ± 0.63	−0.207	0.039
Monocyte count (×10^6^/L)	322.29 ± 102.31	374.65 ± 109.55	−3.500	0.001
TC (mmol/L)	4.66 ± 1.07	4.59 ± 1.14	0.419	0.675
Triglycerides (mmol/L)	1.48 ± 0.84	1.62 ± 1.23	−0.901	0.368
HDL-c (mmol/L)	1.39 ± 0.31	1.26 ± 0.33	2.883	0.004
LDL-c (mmol/L)	2.78 ± 0.83	2.72 ± 0.87	0.494	0.622
MHR	241.35 ± 95.21	316.46 ± 126.09	−4.549	< 0.001
LVDD (mm)	46.77 ± 3.52	49.08 ± 4.71	−4.310	< 0.001
LVEF (%)	67.10 ± 5.68	62.18 ± 5.40	6.481	< 0.001
**Medication use, n (%)**				
Aspirin, n (%)	21.00(30.00)	94.00(47.00)	6.129	0.013
Statins, n (%)	31.00(44.30)	30.00(15.00)	25.429	< 0.001
ACEIs or ARBs, n (%)	15.00(21.40)	37.00(18.50)	0.268	0.593

*PVCs* premature ventricular complexes, *WBC* white blood cell, *TC* total cholesterol, *HDL-c* high-density lipoprotein cholesterol, *LDL-c* low-density lipoprotein cholesterol, *MHR* monocyte to HDL-c ratio, *BMI* body mass index, *LVDD* left ventricular end-diastolic diameter, *LVEF* left ventricular ejection fraction, *ACEI* angiotensin-converting enzyme inhibitor, *ARB* angiotensin receptor blocker

### Comparisons of the monocyte count, MHR, and HDL-c level between the control group and ARS risk-stratified subgroups

**Fig. 1:**
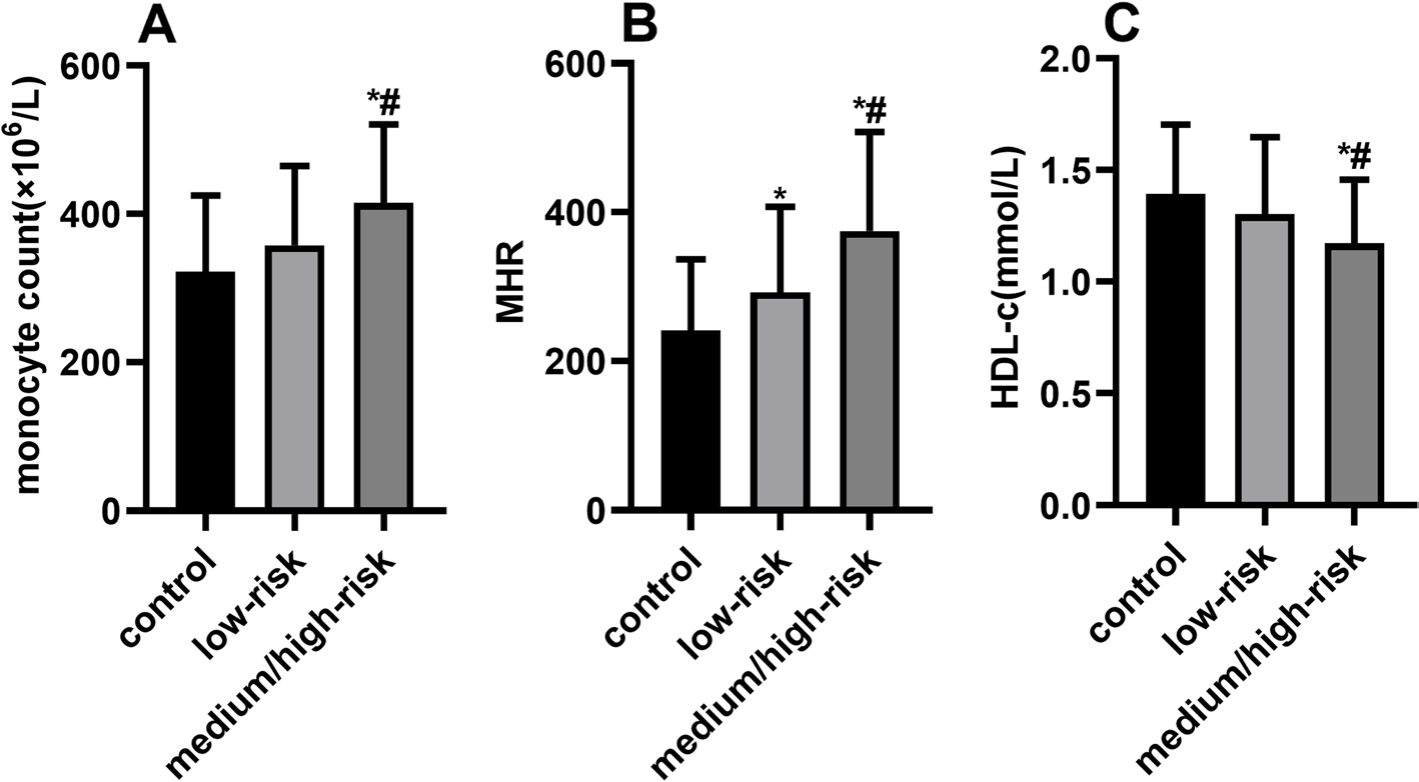
Comparisons of the monocyte count (**A**), MHR (**B**), and HDL-c level (**C**) between the subgroups stratified by axis, burden, coupling interval–ventricular tachycardia (ABC-VT) risk score (ARS) and the control group. In comparation with the control group, *P < 0.05; in comparation with the low-risk subgroup, ^#^*P* < 0.05

The comparisons of the monocyte count, MHR, and HDL-c level between the control group and ARS risk-stratified subgroups are shown in Fig. 1. The monocyte count in the medium−/high-risk subgroup was obviously higher than those in the low-risk and control subgroups. The MHR was the lowest in the control group and highest in the medium−/high-risk subgroup, with that of the low-risk subgroup falling in the middle. In addition, the HDL-c level in the high/medium-risk subgroups was obviously lower than those in the low-risk subgroup and the control groups.

### Correlations of the monocyte count, MHR, and HDL-c level with the ARS

**Fig. 2:**
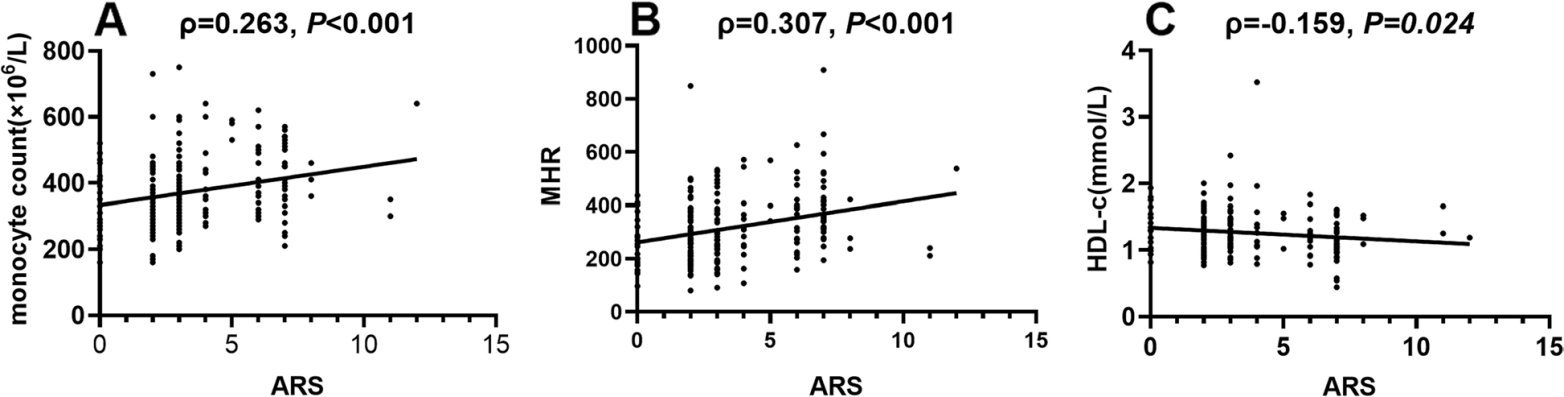
Correlations of the monocyte count (**A**), MHR (**B**) and HDL-c level (**C**) with the ARS

Spearman’s correlation analyses between the monocyte count, MHR, and HDL-c level and the ARS is shown in Fig. 2. The results showed that the ARS was positively related to the MHR (*ρ* = 0.307, *P* < 0.001, Fig. 2B) and monocyte count (*ρ* = 0.263, *P* < 0.001, Fig. 2A) but negatively related to the HDL-c level (*ρ* = − 0.159, *P* < 0.05, Fig. 2C).

### RFs for frequent PVCs

To evaluate the RFs for frequent PVCs, the ARS of the low-risk subgroup was set to 0, and that of the medium−/high-risk subgroup was set to 1 for analysis. Initially, we performed univariate binary logistic regression analyses to identify probable RFs for frequent PVCs, and the variates identified as being significant were analyzed with multivariate binary stepwise logistic regression. WBC, neutrophil, lymphocyte, monocyte counts and the MHR were positively associated with the medium−/high-risk ARS subgroup, whereas HDL-c was negatively associated with the medium−/high-risk ARS subgroup (Table 2). Subsequently, the 6 variates identified as being significant were further studied by multivariate logistic stepwise regression. As shown in Table 3, the MHR was a RF for the medium−/high-risk ARS subgroup [odds ratio = 1.005, 95% confidence interval (CI):1.003–1.008, *P* < 0.001].

**Table 2:** Univariate binary logistic regression analyses of risk factors for frequent PVCs

Variates	Wald χ^**2**^	Standard error	regression coefficient	***P***	Odds ratio (95% confidence interval)
Male sex	2.667	0.313	0.511	0.102	1.667 (0.903,3.007)
Age	3.190	0.010	0.017	0.074	1.018 (0.998,1.037)
BMI	0.045	0.041	0.009	0.832	1.009 (0.931,1.092)
Hypertension	0.416	0.336	0.217	0.519	1.242 (0.643,2.401)
Diabetes	0.966	0.791	−0.777	0.326	0.460 (0.098,2.165)
Smoking	0.831	0.454	0.413	0.362	1.512 (0.622,3.678)
Alcohol consumption	0.026	0.563	0.090	0.873	1.094 (0.363,3.330)
WBC count	6.999	0.104	0.276	0.008	1.317 (1.074,1.616)
Neutrophil count	4.868	0.130	0.288	0.027	1.333 (1.033,1.722)
Lymphocyte count	3.990	0.248	0.495	0.046	1.640 (1.009,2.666)
Monocyte count	10.591	0.001	0.005	0.001	1.005 (1.002,1.008)
Total cholesterol	1.212	0.166	−0.182	0.271	0.833 (0.602,1.153)
Triglycerides	1.781	0.125	0.166	0.182	1.181 (0.925,1.507)
HDL-c	6.412	0.576	−1.459	0.011	0.232 (0.075,0.719)
LDL-c	1.771	0.203	−0.271	0.763	0.763 (0.512,1.137)
MHR	15.131	0.001	0.005	< 0.001	1.005(1.003,1.008)
LVDD	0.424	0.033	−0.022	0.515	0.978 (0.916,1.045)
LVEF	2.590	0.029	−0.047	0.108	0.954 (0.901,1.010)
Aspirin	0.051	0.311	−0.071	0.821	0.932 (0.507,1.714)
Statins	0.249	0.423	0.211	0.618	1.235 (0.539,2.827)
ACEIs or ARBs	0.001	0.399	0.014	0.973	1.014 (0.464,2.214)

Axis, burden, coupling interval–ventricular tachycardia (ABC-VT) risk score (ARS) of medium−/high-risk subgroup = 1; ARS of low-risk subgroup = 0; sex: male = 1, female = 0; alcohol consumption: yes = 1, no = 0; diabetes: yes = 1, no = 0; hypertension: yes = 1, no = 0; smoking: yes = 1, no = 0; aspirin: not using = 1, using = 0; statins: not using = 1, using = 0; ARBs or ACEIs: not using = 1, using = 0; age, BMI, HDL-c, LDL-c, WBC count, monocyte count, LVEF and LVDD were measured values; MHR = monocyte count/HDL-c

**Table 3:** Multivariate binary stepwise logistic regression analyses of risk factors for frequent PVCs (Forward LR)

Variate	Wald χ^**2**^	Standard error	regression coefficient	***P***	Odds ratio (95% confidence interval)
MHR	15.131	0.001	0.005	< 0.001	1.005 (1.003, 1.008)

### ROC curve of the monocyte count, MHR, and HDL-c level to predict the presence of frequent PVCs

**Fig. 3:**
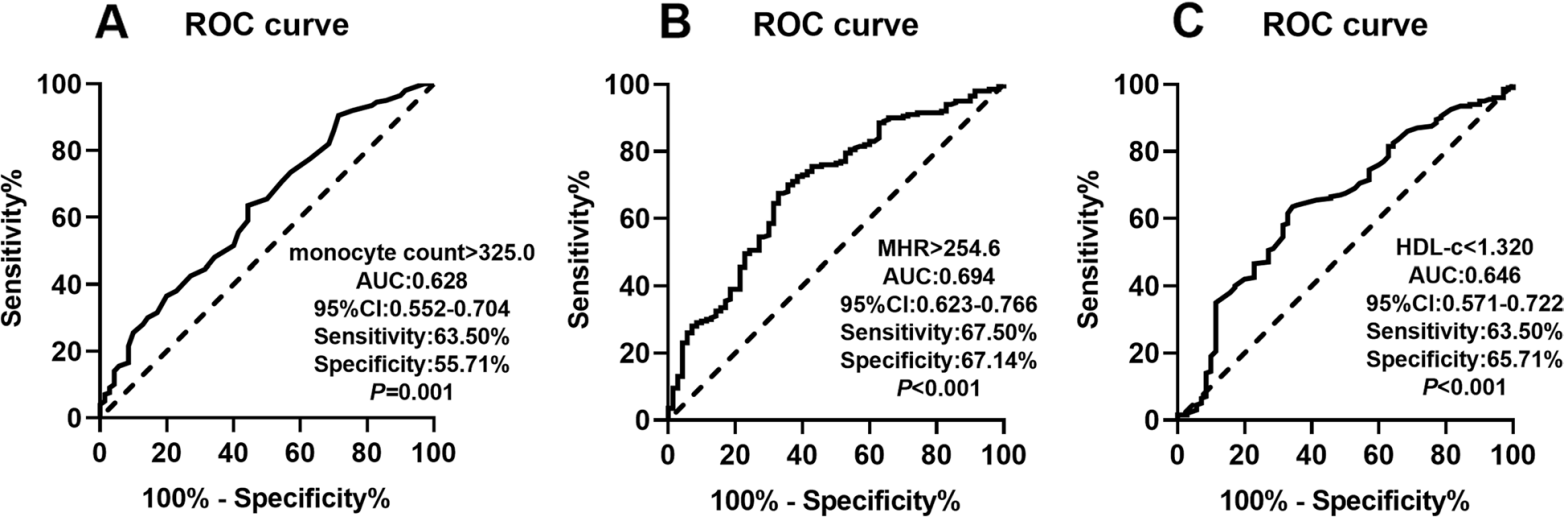
Receiver operating characteristic (ROC) curve of the monocyte count (**A**), MHR (**B**) and HDL-c level (**C**) to predict the presence of frequent PVCs

ROC curve was applied to quantify the ability of the monocyte count, MHR, and HDL-c level to predict frequent PVCs (Fig. 3). A monocyte count cutoff point of 325.0 featured 63.50% sensitivity and 55.71% specificity for predicting frequent PVCs, and the area under the curve (AUC) reached 0.628 (95% CI: 0.552–0.704) (*P* = 0.001). An MHR cutoff point of 254.6 featured 67.50% sensitivity and 67.14% specificity for predicting frequent PVCs, and the AUC reached 0.694 (95% CI: 0.623–0.766) (*P* < 0.001). In addition, an HDL-c level cutoff point of 1.320 featured 63.50% sensitivity and 65.71% specificity for predicting frequent PVCs, and the AUC reached 0.646 (95% CI: 0.571–0.722) (*P* < 0.001).

## Discussion

This investigation discovered that an elevated MHR, considered a reliable inflammatory marker, had an independent statistical relationship with frequent PVCs. In ROC analyses, an MHR > 254.6 determined on admission featured 67.50% sensitivity and 67.14% specificity for predicting frequent PVCs in patients who developed frequent PVCs. The investigation confirmed that an elevated MHR is a RF for persons at medium−/high risk of frequent PVCs and that these patients may be at risk of an adverse prognosis as predicted by the ARS.

PVCs are clinically common arrhythmias. In the sixth century BC, Pien Ts’Io, a Chinese physician, noted that the irregularity frequency could predict mortality [24]. In the late 1800s, Étienne-Jules Marey, a French physiologist and scientist [25], first found that PVCs can result in symptoms such as dizziness or palpitations, but fortuitous PVCs are typically regarded as harmless. Highly frequent PVCs, nonetheless, may damage left ventricular function and can aggravate heart failure [1, 2]. Additionally, PVCs can act as triggers for idiopathic ventricular fibrillation [26].

Recently, the MHR has become a convenient novel marker integrating anti-inflammatory and proinflammatory considerations [16, 27–30]. For example, a study found that the occurrence of cardiac syndrome X had a positive correlation with a higher MHR [31]. Moreover, in persons who underwent coronary angiography, a higher MHR was related to reduced event-free survival and an increased incidence of mainly negative cardiac events [16]. Increased MHR was discovered to have a positive relationship with no reflow [32], stent thrombosis [19], and long-term as well as in-hospital mortality in persons who suffer from myocardial infarction (ST segment elevation in the electrocardiograph) and who underwent percutaneous coronary intervention [33]. Cryoballoon-based catheter ablation is considered a key index for predicting AF recurrence [20]. Nonetheless, little is known about the links between the MHR and frequent PVCs. The current study is the first to reveal that an increased MHR is positively correlated with frequent PVCs and may be associated with an adverse outcome, through ARS prediction.

The precise etiologies of PVCs are still unclear, but the potential mechanisms for any given PVC includes triggered activity, reentry, and automaticity [34]. Studies have found that inflammation is key to arrhythmia progression and development, leading to arrhythmia triggers and reentry [35]. PVC automaticity also has a close connection with inflammation [36]. The MHR, as a novel biomarker of systemic inflammation, may affect the above mechanism. PVCs have been closely linked with myocardial inflammatory conditions [37, 38]. In fact, the production and upregulation of proinflammatory cytokines denote an innate or intrinsic stress response to protect the injured myocardium [39]. In addition, in comparison to healthy participants, circulating proinflammatory cytokines were discovered to be elevated in the sera of young patients with no structural heart disorder who developed ventricular arrhythmias [40]. Based on these studies, Yildiz et al. suggested that the inflammatory course may involve structural ventricular remodeling and electrophysiological changes in relation to PVC development [41]. The MHR may have a similar effect.

Frequent PVCs may cause cardiomyopathy that can be reversed by appropriate PVC inhibition, while some PVCs could lead to sudden death [42]. Hence, it is essential to confirm PVC damage early to guide further treatment options. More recently, it was found that the ARS could predict adverse prognoses of patients who had frequent PVCs [21]. In the British Columbia PVC Registry population, Thibet et al. conducted an external verification of the ARS [22]. The investigation confirmed that an increased MHR was correlated with the ARS to a certain extent and was a risk factor for the medium−/high-risk ARS subgroup; thus, the ARS has certain clinical importance in guiding the treatment strategy for patients with PVCs.

### Comparisons with other studies and what the current work adds to existing knowledge

Nowadays, the MHR was regarded as a novel marker of inflammation. Earlier investigations demonstrated that the MHR was correlated with multiple disorders, especially arrhythmia diseases. What’s more, some investigations have shown that PVCs are closely related to inflammation. However, the relationship between the MHR and frequent PVCs as well as patient prognosis is still unclear. The current investigation demonstrated that an elevated MHR can be key to frequent PVCs occurrence. Moreover, participants with frequent PVCs who had medium−/high MHRs seemed to experience more adverse events.

### Study strengths and limitations

This investigation possesses some strengths. The current investigation demonstrated that the MHR can be used as a practical, cost-saving and simple biomarker of inflammation owing to its value in the prediction of frequent PVCs. Moreover, the current investigation used a new score to evaluate the prognosis of patients with frequent PVCs. The current investigation also has certain limitations. Firstly, this investigation was a simple retrospective study. Secondly, the evaluation of a single sample may not identify variations in the MHR over time.

## Conclusions

In brief, the current investigation showed that the MHR was positively and independently related to frequent PVCs and had value for the prediction of frequent PVCs. These findings imply that the MHR is crucial to prognostic prediction and risk stratification for patients who develop frequent PVCs. These findings increase our knowledge of the links between inflammation and frequent PVCs and offer a candidate for a practical, cost-saving and simple biomarker of inflammation to be collected in the clinic for the management and prevention of frequent PVCs.

## Data Availability

The datasets used and/or analyzed during the current study are available from the corresponding author on reasonable request.

## Abbreviations

ABC-VT: Axis, burden, coupling interval–ventricular tachycardia
ARB: Angiotensin receptor blocker
ACEI: Angiotensin-converting enzyme inhibitor
ARS: ABC-VT risk score
AUC: Area under the curve
AF: Atrial fibrillation
BG: Blood glucose
BP: Blood pressure
BMI: Body mass index
CI: Confidence interval
FAHAMU: The First Affiliated Hospital of Anhui Medical University
ROC: Receiver operating characteristic
HHCH: Hefei High-tech Cardiovascular Hospital
HDL-c: High-density lipoprotein cholesterol
LDL-c: Low-density lipoprotein cholesterol
LVD: Left ventricular end-diastolic
LVDD: LVD diameter
LVEF: Left ventricular ejection fraction
MHR: Monocyte to HDL-c ratio
PVC: Premature ventricular complex
RF: Risk factor
TC: Total cholesterol
WBC: White blood cell
